# Integrated Otolaryngology and Anesthesia Simulation Model for Crisis Management of Cavernous Carotid Artery Injury

**Published:** 2018

**Authors:** Haley E Calcagno, Brandon Lucke-Wold, Michele Noles, Dawn Dillman, Mark Baskerville, Donn Spight, Jeremy N Ciporen

**Affiliations:** 1 Department of Neurological Surgery, Oregon Health & Science University, Portland, OR; 2 Department of Neurosurgery, University of Florida, Gainesville, FL; 3 Department of Anesthesiology and Perioperative Medicine, Oregon Health & Science University, Portland, OR

**Keywords:** ENT Simulation, Anesthesia Simulation, Crisis Management, Teamwork, Skill Development

## Abstract

Simulation training is emerging as a cost-effective way to train residents on the skill sets necessary to excel as fully functioning physicians. Until recently, the simulated resident training environments have primarily focused on handling a medical crisis with learners from the same specialty. A dual otolaryngology and anesthesiology simulation was established to improve teamwork and communication skills between specialties. One otolaryngology resident was paired with one anesthesia resident per trial in our study. The multispecialty team addressed three clinical simulation scenarios to manage a cavernous carotid artery-bleeding crisis with an endoscopic endonasal approach. An independent reviewer evaluated each individual based on situation awareness, decision-making, communications and teamwork, as well as leadership. Residents improved on blood loss, pre and post anatomical exam scores, and communication measures through the course of the scenarios. Residents from both specialties rated the simulation highly and wanted further simulation training in the future. Multidisciplinary simulation training is a novel approach for improving communication skills between specialties prior to entering the wards, clinic, or operative arena. The lessons learned from this multidisciplinary simulation transcend the individual experience by allowing trainees to develop algorithms for crisis management and to improve on aspects of teamwork, leadership, and communication skills that can be applied throughout their careers.

## Introduction

Simulation is an effective method for improving trainee performance in the hospital and operating room settings. Failures in communication and teamwork are frequent in healthcare, and have the potential to result in adverse outcomes for patients^1–3^. While single-specialty simulation has been well established in resident training, recent simulations have begun integrating multidisciplinary surgical and anesthesia components in training courses to more adequately represent real world learning environments. However, to date, these training simulations using multidisciplinary approaches have been limited to commonplace scenarios. Studies by Weller et al. conducted simulations of three clinical events with operating room teams to assess teamwork and communication. After completing the simulation, participants reported lasting changes in collaboration and communication positively affecting clinical practice^1,2^. The simulations lacked the stressors and potential crisis management that is often present in real world medical practice.

In order to effectively manage an intraoperative crisis, trainees must develop the multidisciplinary communication and teamwork skills to coordinate crisis management. Carotid artery injury, although rare, is a serious complication of endoscopic endonasal skull base surgery. It will likely occur at least once during an Otolaryngologists career and requires a calm and practiced approach. We have previously published a multidisciplinary crisis management simulation to train neurosurgery and anesthesia resident teams on management of injury with residents at various levels of expertise. Residents reported learning an algorithm to successfully manage vascular injury, and blood loss during clinical scenarios. Their ability to implement the algorithm improved as residents advanced through the simulation, and anatomical exams improved from pre-to post-simulation^4,5^. Based on these promising initial studies, we expanded the training to otolaryngology to see if improvements can be obtained across different specialties in crisis management.

In this study, we apply our multidisciplinary crisis management simulation to otolaryngology and anesthesia resident teams. We use specialty-specific and team-based debriefing to attempt to improve communication and teamwork through 3 clinical scenarios. The debriefing was formulated to allow open communication and long-term learning. The results of this study are promising in that the reliability of the training environment has been maintained across specialties.

## Methods

### Study Design

All simulations were approved by the Oregon Health & Science University (OHSU) Institutional Review Board, and completed in the Virtu OHSU laboratory. This resident-training simulation consisted of three scenarios necessitating crisis management in the setting of cavernous carotid artery injury, each increasing in complexity and requiring more advanced levels of communication. The three scenarios each involved varied patient profiles and implications for management, as described previously^5^. Briefly, the three scenarios included cavernous carotid bleed in a healthy patient, a patient with coronary artery disease, and a patient on chronic steroids. Each resident team consisted of one otolaryngology and one anesthesia resident collaborating through the three scenarios, with a total of six otolaryngology and six anesthesia residents completing the course (n=12 learners). Prior to each scenario, respective faculty provided instructions regarding the simulation (detailed below). During all scenarios, blood loss values were recorded, and independent faculty evaluators rated resident performance as described below. After each scenario, residents were provided with a debrief session on their performance and areas for improvement. Otolaryngology residents completed a pre-simulation anatomical exam to assess baseline knowledge, and were retested after completion of the simulation.

### Simulation Preparation

Setup of the simulation included a Laerdal SimMan (Laerdal Medical, Wappingers Falls, NY), prepared cadaver head connected to vascular perfusion pump, arterial line with pressure transducer, patient monitor, and laptop control computers. An intravenous (IV) pole, IV bags with tubing, and functional IV units were available. Otolaryngology residents were provided with an instrument table, stool, and operating room (OR) drapes. Anesthesia residents were provided with a cart containing a drug tray, an anesthesia machine, and anesthesia monitor.

### Cadaveric Set-up

Preparation of the cadaveric head was similar to prior publication^4^. Briefly, exposure of the sphenoid sinus was performed endoscopically. The right cavernous carotid artery was exposed and an incision made at the level of the genu to serve as the bleed site. During the simulation, the right common carotid artery was cannulated, secured by clamp, and infused with simulated blood via a rapid infusion Belmont pump (rate in mL/min). Mean arterial pressure (MAP) produced by infusion were measured via arterial line and were set to mimic a typical operating room experience based on the three clinical scenarios. Mean arterial pressures spanned a range of 65–110 mmHg during the simulations.

### Otolaryngology Instructions

Neurosurgery faculty provided otolaryngology residents with instructions on surgical technique and anatomy prior to beginning the simulation. Each otolaryngology resident was paired with an anesthesia resident to complete the three 10 minute scenarios. Learning objectives for otolaryngology residents included: 1) appropriately communicate case events and goals for management, 2) develop an algorithm for managing cavernous carotid injury in varied clinical scenarios, and 3) coordinate management of blood pressure during scenarios necessitating disparate medical management, and prioritizing crisis management versus long term implications for the patients. After the completion of each scenario, neurosurgery faculty provided the otolaryngology learners with a 5-minute debrief of performance feedback and areas for improvement on technical skill and teamwork. After specialty-specific feedback, both otolaryngology and anesthesia residents joined for a 5 minute inter-professional debrief session.

### Anesthesia Instructions

Prior to the simulation, anesthesia faculty provided residents with instructions on use of the anesthesia monitor, and description of presented clinical scenarios. Each anesthesia resident was paired with an otolaryngology resident, as described above. Objectives for anesthesia learners included 1) coordinate care of a patient with carotid bleeding, 2) use at least three different communication strategies, 3) allocate resources for the management of the crisis, 4) manage blood pressure and blood loss for each different scenario, and 5) manage coronary ischemia in the setting of blood loss and reduced blood pressure. Scenarios were followed by faculty-led debriefs as described above (5 min specialty-specific and 5 min inter-professional).

### Operative Environment

Each resident team was provided with the same endoscopic dissection, bony exposure, and anesthesia setup, and utilized a 4-handed surgical approach. A faculty member provided relevant anatomical orientation when needed, and the endoscope during all simulations to standardize visualization between all resident teams. Otolaryngology learners controlled all operative instruments.

Otolaryngology and anesthesia residents collaborated to control blood pressure, blood loss, and gain vascular control. Pressure and a half-cottonoid patty were utilized to gain initial vascular control of the carotid bleed, which was then replaced with a muscle graft from the temporalis muscle.

### Resident Assessment & Feedback

During the simulation, resident performance was evaluated on a 4-point scale (1-poor, 2-marginal, 3-acceptable, 4-good) based on situational awareness, decision-making, communications and teamwork, and leadership. Scores were averaged for each specialty and reported as the mean ± standard error of the mean (SEM). A t-test was used to evaluate between specialties, and results considered statistically significant when p<0.05.

**Figure 1:**
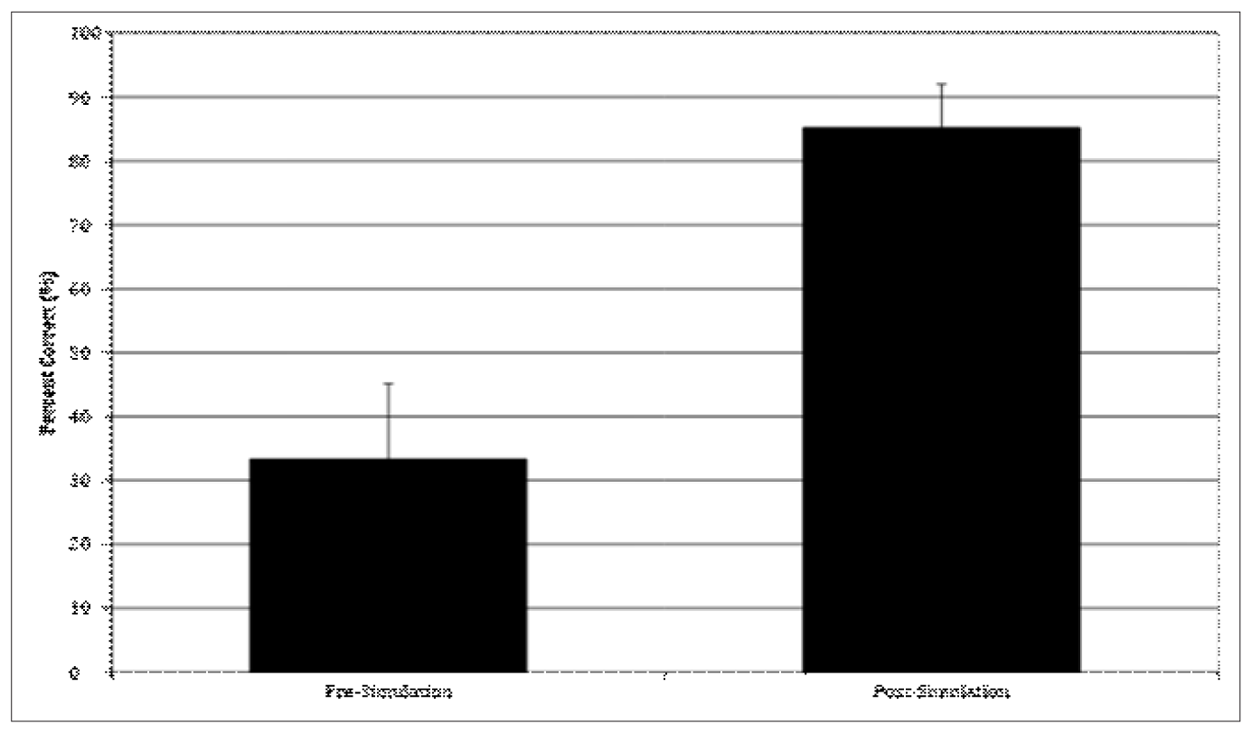
Overall resident performance on anatomical test performance pre (33.33 ± 11.83) and post (85.19 ± 6.83) simulation (n = 6 otolaryngology residents). Data presented as mean ± SEM

Otolaryngology residents completed an anatomical exam pre and post-simulation to evaluate for pre-simulation knowledge and improvement. Exams consisted of an endoscopic image with questions indicated by labeled lines (exam and key detailed in Figure 1). Average percentage correct scores on anatomical exams were compared pre and post-simulation. All residents were given post-simulation surveys to evaluate skill improvement and applicability of the simulation. Anesthesia residents completed an additional survey relevant to the realism of the anesthesia model and teaching feasibility.

## Results

### Resident Performance

Blood loss for each resident team was recorded for each scenario and is presented in Table 1. Mean team blood loss improved over the three scenarios (1100 ± 146.06 mL scenario 1, 716.76 ± 90.06 mL scenario 2, 475 ± 91.06 mL scenario 3). Otolaryngology residents significantly improved on gathering information (p<0.05) and tended towards improvement on exchanging information (p=0.055) across the 3 scenarios (Table 2). Anesthesia residents significantly improved on gathering information (p<0.05) and exchanging information (p<0.05) across the 3 scenarios (Table 3). Both otolaryngology and anesthesia residents scored highly (above a score of 3 on all scenarios) for the ‘coping with pressure’ measure. Senior otolaryngology residents (PGY 5) performed slightly better than Junior (PGY 3) otolaryngology residents, but this did not reach statistical significance (Table 4). Senior residents most highly outscored Junior residents on ‘selecting and communicating options’ and ‘communicating options’ (average score difference=1.23, 1.11 respectively). Junior residents consistently improved on the ‘situational awareness’ parameters where the senior residents did not, which trends towards statistical significance (p=0.08, Table 4). Junior residents scored similarly to Senior resident performance on scenarios 1 and 2, and higher on scenario 3 for ‘projecting and anticipating future states’ (average score difference=−0.33).

### Anatomical Exam

Prior to the simulation, otolaryngology residents completed an anatomy exam to assess for knowledge of relevant skull base anatomy. After the simulation, residents completed an identical anatomical exam (Table 5). Residents scored an average of 33.33% correct on the pre-simulation exam, and improved to an average of 85.19% correct post-simulation, with all residents showing score improvement from pre to post (Figure 1). The identifications missed most frequently pre-simulation were the face of the sphenoid (0% correct), tuberculum sellae (0% correct for all residents), and clivus (16.7% correct for all residents), and identification of these structures improved post-simulation (face of sphenoid 66.7% correct, tuberculum sellae 83.3% correct, clivus 66.7% correct for all residents). After the simulation, all otolaryngology residents reported the simulation improved or reinforced their knowledge of relevant anatomy (Table 6).

### Resident Feedback

Resident post-simulation feedback by specialty is reported in Table 6. After completion of the simulation, all otolaryngology residents were more comfortable using endoscopic instrumentation, and agreed the simulation improved their surgical skill set. Some otolaryngology residents gave feedback that further labs before beginning the scenarios would be useful in future simulations. Both otolaryngology and anesthesia residents found the simulation valuable, felt the lessons experienced can be translated to other crisis scenarios, and all would participate in simulation again if offered in the future. Most importantly, all residents reported developing an algorithm for managing carotid artery injury. In addition, anesthesia residents completed a set of questions unique to their role in the simulation (Table 7). All anesthesia residents agreed the simulation was clinically applicable to their field, and will help them in future similar situations and to prevent errors. Some anesthesia residents requested more time to get familiar with the mannequin.

## Discussion

Our simulation model trains residents on crisis management and incorporates the communication and teamwork skills directly translatable to the operative setting. Otolaryngology residents reported improved or reinforced anatomical knowledge due to the simulation, as supported by increased pre- to post-simulation anatomy exam scores. Simulation resulted in improved team performance, resulting in reduced blood loss and improved ‘gathering information’ for both specialties across the scenarios. Both otolaryngology and anesthesia residents improved on gathering information across the scenarios, and otolaryngology trended towards improvement and anesthesia residents improved on exchanging information. This suggests that the simulation scenarios may help improve communication in the peri-operative arena, which is a critical skill for training. Although senior residents scored slightly higher than junior residents on faculty-rated performance parameters, this was not statistically significant. Furthermore, junior residents consistently improved on situational awareness, where the senior residents did not.

Of note, there was a wide range in the perceived stress level during the training and debriefing process, and there was wide variability in the preferred environment for learning. This is an important aspect of our simulation, as residents need to adapt to learning in multiple different environments requiring them to venture out of their comfort zone. Future studies will look at how this perceived stress influences performance. The use of biometric data obtained from residents during the training session such as heart rate and blood pressure will aid in correlating stress to performance in future simulations. The pre-training and debriefing sessions will focus on stress management and appropriate response in times of crisis. By using biometric monitors throughout the simulation, the faculty instructors can help train healthy stress management approaches during the debriefing session and encourage implementation of these approaches in real world settings.

None of them could objectively demonstrate that skills acquired from simulation are transferred to the operating theatre or show a demonstrable benefit in patient outcomes none of them could objectively demonstrate that skills acquired from simulation are transferred to the operating theatre or show a demonstrable benefit in patient outcomes none of them could objectively demonstrate that skills acquired from simulation are transferred to the operating theatre or show a demonstrable benefit in patient outcomes Although published multidisciplinary simulations report improved skill parameters after simulation and positive reactions from participants, no studies are currently able to connect skills developed within simulation to improved performance in practice^3^. Another step for future studies is to assess the impact of multidisciplinary training simulations on operative management and patient outcomes. We are going to track resident performance over time by patient outcome measurements for crisis management, yearly surveys, and simulation improvement scores by year. Additionally, faculty involvement will be obtained to assess resident attainment of milestones. Additional crisis management training modules are being developed in order to expose residents to new clinically relevant learning environments. One of the key elements will be applicability to the real world, which will be facilitated by faculty trainers. Faculty feedback to residents in real life clinical scenarios can draw back to the simulations and will help cement ideas and skills learned during the simulation. Another added benefit is the ability to conduct impromptu 5-minute debriefings throughout the academic year to discuss communication strategies similar to the debriefings used in the simulation module.

The ultimately goal is improved patient care and safety. To achieve this, we are incorporating the help of resident team leaders to help improve the simulation modules. This quality improvement initiative allows residents to take an active role in designing scenarios that they both see frequently clinically and also need extra guidance for management. By laying the groundwork in this crisis management study, residents have a framework upon which to build their ideas for quality improvement projects. It is our goal to expand the initiative across the entire university by championing resident led simulation encounters.

In summary, crisis management simulation is an extremely valuable learning tool to train residents. We have shown that multidisciplinary learning has real-world practicality, enhances communication, and is linked to measurable improvements. Going forward we will expand the training in order to facilitate improved patient care. We are pioneering several initiatives towards this goal as outlined in the discussion above.

## Figures and Tables

**Table 1:** Resident group blood volume loss across 3 clinical scenarios

Group	Scenario 1 (10 min)	Scenario 2 (10 min)	Scenario 3 (10 min)
1	1300 mL	1100 mL	900 mL
2	1100 mL	550 mL	500 mL
3	1600 mL	750 mL	400 mL
4	600 mL	800 mL	400 mL
5	1200 mL	500 mL	250 mL
6	800 mL	600 mL	400 mL
Mean ± SEM	1100 ± 146.06 mL	716.67 ± 90.06 mL	475 ± 91.06 mL

**Table 2:** Otolaryngology average group performance across 3 clinical scenarios (n = 6 otolaryngology residents). Ratings are on a 1–4 scale based on faculty observation

Category	Element	Average Score	Average Score	Average Score	Two-Way
		Scenario 1	Scenario 2	Scenario 3	ANOVA
**Situation Awareness**	Gathering Information	2.00	2.50	3.00	F(2,14)=7.5, p<0.01
	Understanding Information	2.70	3.20	3.00	F(2,14)=1.3, p>0.05
	Projecting and anticipating future state	2.70	2.70	3.20	F(2,14)=3.4, p>0.05
**Decision Making**	Considering options	2.50	2.50	3.00	F(2,15)=0.5, p>0.05
	Selecting and communicating options	2.50	3.00	3.20	F(2,15)=0.6, p>0.05
	Implementing and reviewing decisions	2.30	2.80	3.70	F(2,15)=1.4, p>0.05
**Communications and Teamwork**	Exchanging information	2.30	3.20	3.70	F(2,15)=3.6, p>0.05
	Establishing a shared understanding	2.30	2.80	3.00	F(2,14)=3.3, p>0.05
	Coordinating team activities	2.50	2.50	2.50	F(2,14)=0.5, p>0.05
**Leadership**	Setting and maintaining standards	2.20	2.70	2.70	F(2,14)=1.9, p>0.05
	Supporting others	2.30	2.30	2.30	F(2,13)=2.7, p>0.05
	Coping with pressure	3.30	3.70	3.20	F(2,14)=1.3, p>0.05

Scoring scale:

1 Poor Performance endangered or potentially endangered patient safety, serious remediation is required

2 Marginal Performance indicated cause for concern, considerable improvement is needed

3 Acceptable Performance was of satisfactory standard but could be improved

4 Good Performance was of a consistently high standard, enhancing patient safety; it could be used as a positive example for others

**Table 3:** Anesthesia average group performance across 3 clinical scenarios (n = 6 anesthesia residents). Ratings are on a 1–4 scale based on faculty observation

Category	Element	Average Score	Average Score	Average Score	Two-Way
		Scenario 1	Scenario 2	Scenario 3	ANOVA
**Situation Awareness**	Gathering Information	2.33	2.17	3.50	F(2,15)=4.1, p<0.05
	Understanding Information	2.33	2.67	3.33	F(2,15)=1.5, p>0.05
	Projecting and anticipating future state	2.33	2.33	3.33	F(2,15)=1.5, p>0.05
**Decision Making**	Considering options	2.33	3.00	3.00	F(2,15)=1.8, p>0.05
	Selecting and communicating options	2.83	3.00	3.33	F(2,15)=0.7, p>0.05
	Implementing and reviewing decisions	2.50	2.50	3.17	F(2,15)=1.5, p>0.05
**Communications and Teamwork**	Exchanging information	2.50	3.00	3.83	F(2,15)=4.0, p<0.05
	Establishing a shared understanding	2.50	3.00	3.67	F(2,15)=2.8, p>0.05
	Coordinating team activities	2.17	2.50	3.17	F(2,15)=3.3, p>0.05
**Leadership**	Setting and maintaining standards	2.17	2.33	3.17	F(2,15)=2.3, p>0.05
	Supporting others	2.33	2.67	3.33	F(2,15)=1.7, p>0.05
	Coping with pressure	3.33	3.67	3.67	F(2,15)=0.8, p>0.05

Scoring scale:

1 Poor Performance endangered or potentially endangered patient safety, serious remediation is required

2 Marginal Performance indicated cause for concern, considerable improvement is needed

3 Acceptable Performance was of satisfactory standard but could be improved

4 Good Performance was of a consistently high standard, enhancing patient safety; it could be used as a positive example for others

**Table 4:** Otolaryngology resident average performance based on Junior (PGY 3) or Senior (PGY 5) status by clinical scenario (S). Ratings are on a 1–4 scale based on faculty observation

Category	Question	PGY 3 Scores (N=3)			PGY 5 Scores (N=3)			
		S 1	S 2	S 3	S 1	S 2	S 3	
**Situation Awareness**	Gathering Information Understanding	1.33	2.33	3.67	2.67	2.67	2.33	
	Information Projecting and anticipating future state	2.33	2.67	4.00	3.00	3.67	2.67	
	Projecting and anticipating future state	2.33	2.67	4.00	3.00	2.67	2.33	
	Average	2.00	2.56	3.67	2.89	3.00	2.44	F(2,3) = 6.88, p=0.08
**Decision Making**	Considering options	1.67	2.00	2.33	3.00	3.00	3.33	
	Selecting and communicating options	2.00	2.33	2.33	3.00	3.67	3.67	
	Implementing and reviewing decisions	2.33	2.33	2.67	2.67	3.33	3.67	
	Average	2.00	2.67	3.67	2.89	3.33	3.56	F(2,3) = 0.09, p=0.91
**Communications and Teamwork**	Exchanging information	2.00	2.67	3.67	2.67	3.67	3.67	
	Establishing a shared understanding	2.33	1.67	2.67	2.33	3.33	2.67	
	Coordinating team activities	2.33	1.67	2.67	2.67	3.33	2.33	
	Average	2.22	2.22	3.22	2.56	3.44	2.89	F(2,3) = 1.35, p=0.38
**Leadership**	Setting and maintaining standards	1.67	2.33	2.00	2.67	3.00	3.33	
	Supporting others	2.33	1.67	2.00	2.33	3.00	2.67	
	Coping with pressure	3.33	3.33	2.33	3.33	4.00	4.00	
	Average	2.44	2.44	2.11	2.78	3.33	3.33	F(2,3) = 1.87, p=0.29

Scoring scale:

1 Poor Performance endangered or potentially endangered patient safety, serious remediation is required

2 Marginal Performance indicated cause for concern, considerable improvement is needed

3 Acceptable Performance was of satisfactory standard but could be improved

4 Good Performance was of a consistently high standard, enhancing patient safety; it could be used as a positive example for others

**Table 5:** Individual question performance on anatomical test pre and post simulation (n = 6 otolaryngology residents)

Question	Resident 1		Resident 2		Resident 3		Resident 4		Resident 5		Resident 6	
Identify	Pre X	Post	Pre	Post	Pre	Post	Pre	Post	Pre	Post	Pre	Post
Right sphenoid ostia/sinus	X	√	√	√	√	√	X	√	X	X	X	X
Left sphenoid ostia/sinus	X	√	√	√	√	√	X	√	X	√	X	X
Face of sphenoid	X	√	X	√	X	√	X	√	X	X	X	X
Tuberculum sellae	X	X	X	√	X	√	X	√	X	√	X	√
Left optic nerve	X	√	√	√	√	√	X	√	√	√	X	√
Left optico carotid recess	X	√	√	√	√	√	√	√	√	√	√	√
Sella turcica/pituitary	X	√	X	√	√	√	X	√	√	√	X	√
Clivus	X	√	X	X	X	√	X	√	√	√	X	X
Right cavernous carotid artery	X	√	√	√	√	√	X	√	√	√	X	√
% Correct	0	88.9	55.6	88.9	66.7	100	11.1	100	55.6	77.8	11.1	55.6

Scoring Scale: X = incorrect answer √ = correct answer

**Table 6:** Resident post-simulation survey feedback based on specialty. Survey response options included Strongly Disagree, Disagree, Neutral, Agree, and Strongly Agree. N/A indicates question is inapplicable to specialty

	Question:	Otolaryngology (n=6)	Anesthesia (n=6)
1.	Did you find the simulation session valuable?	100% Strongly Agree (n=6)	66.7% Strongly Agree (n=4) 33.3% Agree (n=2)
2.	Did you develop an algorithm for managing carotid artery injury?	100% Strongly Agree (n=6)	33.3% Strongly Agree (n=2) 66.7% Agree (n=4)
3.	Do you feel more comfortable using endoscopic instrumentation?	33.3% Strongly Agree (n=2) 66.7% Agree (n=4)	N/A
4.	Do you feel comfortable working with residents from other specialties?	66.7% Strongly Agree (n=4) 33.3% Agree (n=2)	50% Strongly Agree (n=3) 50% Agree (n=3)
5.	Is simulation a good complement to operative experience?	83.3% Strongly Agree (n=5) 16.7% Agree (n=1)	66.7% Strongly Agree (n=4) 33.3% Agree (n=2)
6.	Simulation improved my surgical skill set	83.3% Strongly Agree (n=5) 16.7% Agree (n=1)	N/A
7.	Do you feel comfortable with crisis resource management?	16.7% Strongly Agree (n=1) 83.3% Agree (n=5)	66.7% Agree (n=4) 33.3% Neutral (n=2)
8.	Would you like to see further similar simulated experiences offered in the future?	66.7% Strongly Agree (n=4) 33.3% Agree (n=2)	66.7% Strongly Agree (n=4) 33.3% Agree (n=2)
9.	Can the lessons learned/experienced in the simulation be translated to other crisis scenarios?	66.7% Strongly Agree (n=4) 33.3% Agree (n=2)	66.7% Strongly Agree (n=4) 33.3% Agree (n=2)
10.	The simulation model offers benefits not available in existing training models	66.7% Strongly Agree (n=4) 33.3% Agree (n=2)	50% Strongly Agree (n=3) 33.3% Agree (n=2) 16.7% Neutral (n=1)
11.	Was this course valuable in your training experience?	83.3% Strongly Agree (n=5) 16.7% Agree (n=1)	66.7% Strongly Agree (n=4) 33.3% Agree (n=2)
12.	Skills learned in the simulated environment translate to the operating room	83.3% Strongly Agree (n=5) 16.7% Agree (n=1)	66.7% Strongly Agree (n=4) 33.3% Agree (n=2)
13.	Simulated experiences should be incorporated into surgical training prior to entering the operating room	66.7% Strongly Agree (n=4) 16.7% Agree (n=1) 16.7% Disagree (n=1)	16.7% Strongly Agree (n=1) 33.3% Agree (n=2) 33.3% Neutral (n=2) 16.7% Strongly Disagree (n=1)
14.	Simulation improved/reinforced my understanding of the relevant anatomy	83.3% Strongly Agree (n=5) 16.7% Agree (n=1)	N/A
15.	Would you like to see simulation integrated into the curriculum?	66.7% Strongly Agree (n=4) 33.3% Agree (n=2)	50% Strongly Agree (n=3) 50% Agree (n=3)
16.	If given the choice, would you participate in simulation in the future?	66.7% Strongly Agree (n=4) 33.3% Agree (n=2)	50% Strongly Agree (n=3) 50% Agree (n=3)

**Table 7:** Anesthesia model survey feedback (n = 6 anesthesia residents). Survey response options included Strongly Disagree, Disagree, Slightly Disagree, Neutral, Slightly Agree, Agree, and Strongly Agree

**Realism**	The simulation was realistic	16.7% Strongly Agree (n=1) 33.3% Agree (n=2) 16.7% Slightly Agree (n=1) 33.3% Neutral (n=2)
	The scenario was clinically applicable to my field	50% Strongly Agree (n=3) 50% Agree (n=3)
	This type of scenario exists in real life	33.3% Strongly Agree (n=2) 33.3% Agree (n=2) 33.3% Slightly Agree (n=2)
	The mannequin is realistic	16.7% Strongly Agree (n=1) 16.7% Agree (n=1) 50% Slightly Agree (n=3) 16.7% Neutral (n=1)
	The environment is realistic	16.7% Strongly Agree (n=1) 66.7% Slightly Agree (n=4) 16.7% Neutral (n=1)
	The actors were realistic	33.3% Strongly Agree (n=2) 16.7% Agree (n=1) 50% Slightly Agree (n=3)
	The physiologic response to my actions was realistic	16.7% Strongly Agree (n=1) 50% Agree (n=3) 16.7% Slightly Agree (n=1) 16.7% Slightly Disagree (n=1)
	The monitor was easy to use	33.3% Strongly Agree (n=2) 33.3% Agree (n=2) 33.3% Slightly Agree (n=2)
**Teaching**	The scenario was clinically applicable to my field	50% Strongly Agree (n=3) 50% Agree (n=3)
	There was sufficient time to perform critical actions	33.3% Strongly Agree (n=2) 66.7% Agree (n=4)
	I had time to get familiar with the mannequin	33.3% Slightly Agree (n=2) 33.3% Neutral (n=2) 16.7% Slightly Disagree (n=1) 16.7% Disagree (n=1)
	This simulation will help me in future similar situations	50% Strongly Agree (n=3) 33.3% Agree (n=2) 16.7% Slightly Agree (n=1)
	The debriefing (if applicable) was stressful	16.7% Agree (n=1) 16.7% Slightly Agree (n=1) 16.7% Neutral (n=1) 16.7% Slightly Disagree (n=1) 33.3% Disagree (n=2)
	I learned something new today	66.7% Strongly Agree (n=4) 33.3% Agree (n=2)
	Simulation is a useful tool	66.7% Strongly Agree (n=4) 16.7% Agree (n=1) 16.7% Slightly Agree (n=1)
	The staff were effective facilitators in the debriefing	50% Strongly Agree (n=3) 33.3% Agree (n=2) 16.7% Slightly Agree (n=1)
	This simulation will help me prevent errors	50% Strongly Agree (n=3) 33.3% Agree (n=2) 16.7% Slightly Agree (n=1)
	I prefer my usual clinical setting for teaching	16.7% Strongly Agree (n=1) 16.7% Agree (n=1) 33.3% Slightly Agree (n=2) 16.7% Neutral (n=1) 16.7% Strongly Disagree (n=1)
	I learned something today I will share with my colleagues	33.3% Strongly Agree (n=2) 33.3% Agree (n=2) 16.7% Slightly Agree (n=1) 16.7% Slightly Disagree (n=1)
	This simulation reviewed/taught me something useful	33.3% Strongly Agree (n=2) 50% Agree (n=3) 16.7% Slightly Agree (n=1)
**General**	Simulation helps me practice for real life	66.7% Strongly Agree (n=4) 16.7% Agree (n=1) 16.7% Neutral (n=1)
	I have used principles from previous simulations in real life	66.7% Strongly Agree (n=4) 33.3% Slightly Agree (n=2)
	Simulation has added to my education	66.7% Strongly Agree (n=4) 16.7% Agree (n=1) 16.7% Slightly Agree (n=1)
	Teaching using simulation does enhance my program	66.7% Strongly Agree (n=4) 16.7% Agree (n=1) 16.7% Slightly Agree (n=1)
	The center was well organized	33.3% Strongly Agree (n=2) 50% Agree (n=3) 16.7% Slightly Agree (n=1)
	I would like to return to this center	50% Strongly Agree (n=3) 16.7% Agree (n=1) 16.7% Slightly Agree (n=1) 16.7% Neutral (n=1)
